# Characterization of Tail Sheath Protein of N4-Like Phage phiAxp-3

**DOI:** 10.3389/fmicb.2018.00450

**Published:** 2018-03-15

**Authors:** Zheng Zhang, Changyu Tian, Jiangtao Zhao, Xiao Chen, Xiao Wei, Huan Li, Weishi Lin, Ruo Feng, Aimin Jiang, Wenhui Yang, Jing Yuan, Xiangna Zhao

**Affiliations:** ^1^College of Food Science, South China Agricultural University, Guangzhou, China; ^2^Institute of Disease Control and Prevention, China PLA, Beijing, China; ^3^Department of Histology and Embryology, School of Basic Medical Sciences, Zhengzhou University, Zhengzhou, China; ^4^State Key Laboratory of Pathogen and Biosecurity, Beijing Institute of Microbiology and Epidemiology, Beijing, China

**Keywords:** *Achromobacter xylosoxidans* A22732, tail sheath protein, phage adsorption assay, enzyme-linked immunosorbent assay (ELISA), characterization

## Abstract

*Achromobacter* phage phiAxp-3, an N4-like bacteriophage, specifically recognize *Achromobacter xylosoxidans* lipopolysaccharide (LPS) as its receptor. PhiAxp-3 tail sheath protein (TSP, ORF69) shares 54% amino acid sequence identity with the TSP of phage N4 (gp65); the latter functions as a receptor binding protein and interacts with the outer membrane receptor NfrA of its host bacterium. Thus, we hypothesized that ORF69 is the receptor-binding protein of phiAxp-3. In the present study, a series of ORF69 truncation variants was constructed to identify the part(s) of this protein essential for binding to *A. xylosoxidans* LPS. Phage adsorption and enzyme-linked immunosorbent assay showed that amino acids 795–1195 of the TSP, i.e., ORF69(795–1195), are sufficient and essential for receptor and binding. The optimum temperature and pH for the functions of ORF69 and ORF69(795–1195) are 4/25°C and 7, respectively. *In vitro* cytotoxicity assays showed that ORF69 and ORF69(795–1195) were respectively toxic and non-toxic to a human immortalized normal hepatocyte cell line (LO2; doses: 0.375–12 μg). The potential of this non-toxic truncated version of phiASP-3 TSP for clinical applications is discussed.

## Introduction

Given their rapid proliferation and high specificity, phages are extensively used in several applications, including rapid detection of pathogenic bacteria (Schofield and Westwater, 2009), assessment of water quality and sewage treatment efficiency (Durán et al., 2002), and classification and identification of bacterial species (Richter et al., 2017). These applications of phages are generally based on the premise that the tail protein can identify receptors on the bacterial surface. Moreover, phage-based disease treatments have attracted recent research attention because of increasing concerns with drug resistance in bacteria (Park and Myung, 2014; Hodyra-stefaniak et al., 2015; Reardon, 2017). The tail protein of phages is significant for the interaction between bacteriophages and host bacteria, and this interaction is a research topic with considerable potential. Singh et al. (2010) have shown the application of genetically engineered bacteriophage tail proteins as molecular probes for the sensitive and selective detection of *Salmonella enterica*. Miernikiewicz et al. (2016) used the tail protein gp12 of T4 phage *in vivo* in rice to counteract lipopolysaccharide (LPS)-induced inflammation.

Recognition of and connection with host cells are important steps for all phages to infect bacterial cells. In general, phages can only be stably adsorbed onto the host bacterial surface by identifying an exposed structure on the cell surface as the recognition receptor and then injecting their genetic materials into the cytoplasm of the host bacteria (Krüger and Schroeder, 1981; São-José et al., 2004). Porins, affinity transporters, and LPS are the most common outer membrane components that act as receptors for tailed phages that infect Gram-negative bacterium (Lindberg, 1973; São-josé et al., 2006). N4 phage is one type of phage that infects the Gram-negative bacterium *Escherichia coli*. The protein gp65, which constitutes a sheath surrounding the tail tube of N4 phage (Choi et al., 2008), is adsorbed onto the cell surface by recognition of the outer membrane protein NfrA (Kiino and Rothmandenes, 1989; Kiino et al., 1993; Mcpartland and Rothman-Denes, 2009). Mcpartland and Rothman-Denes (2009) speculated that direct interaction of NfrA with gp65 is responsible for the initial signaling event leading to N4 DNA and vRNAP injection. *Achromobacter xylosoxidans* A22732 is a Gram-negative bacterium that was isolated from a Chinese patient with pneumonia and is resistant to multiple β-lactam antibiotics, including carbapenems. This dangerous opportunistic pathogen is distributed in the water environment (Sader and Jones, 2005; Chen et al., 2014). Previous research identified the N4-like phage phiAxp-3 in hospital wastewater. This phage specifically recognizes and pyrolyzes *A. xylosoxidans* A22732 and therefore has potential for phage therapy. The genome of phiAxp-3 has been fully sequenced and annotated to determine whether there were any potentially “undesirable” genes (e.g., virulent and/or toxic genes) present (Ma et al., 2016). The absence of these “undesirable” genes has important safety implications (Soffer et al., 2017). No toxin, virulence, repressor genes, integrases, recombinases nor any bacterial gene were detected (Ma et al., 2016). The genome analyses in previous study provide no evidence of any potentially dangerous transducing ability of this lytic phage, supporting the potential of the phage for biocontrol applications (Ma et al., 2016). Experimental results on receptor identification showed that the receptor of phiAxp-3 is LPS (Ma et al., 2016). LPS can trigger inflammatory responses in humans and animals. Analysis of the interactions between the tail proteins of phages and the LPS receptor can be used to develop potential drugs for clinical treatment (Miernikiewicz et al., 2016).

In this study, tail sheath protein (TSP) sequences of phiAxp-3 were screened bioinformatically, and a region with the same effect as the full-length TSP of the phage was identified by protein truncation experiments. The characteristics of the truncated protein, including toxicity, were investigated. The results of this study offer theoretical foundations for future applications of the TSP of N4-like phages.

## Materials and Methods

### Bacterial Strains, Phages, Plasmids, and Media

*Achromobacter xylosoxidans* A22732 and phiAxp-3 were stored in our laboratory and cultured at 37°C. *E. coli* DH5α, *E. coli* BL21 (»DE3) competent cells and protein expression plasmid pET28a were purchased from Beijing BIOMED, Co., Ltd. Luria-Bertani (LB) medium was used for bacterial liquid cultures, and soft-agar medium included an additional 0.7% (wt./vol.) agar.

### Cloning of TSP Genes of phiAxp-3

A 25 μL polymerase chain reaction (PCR) system was established by using phiAxp-3 genomic DNA. **Table 1** lists the primers used. PCR products were recycled using the QIAquick Gel Extraction Kit. *Nhe*I and *Bam*HI double enzyme digestion was performed on PCR products and carrier plasmids. DNA fragments were ligated overnight at 4°C and the target segments of the TSP gene were cloned into pET28a.

**Table 1:** Relevant information on clones produced and investigated in this work.

Protein expression	Primer F (NheI)/R (BamHI)	Location (bp)	Additive amount of IPTG (mM/L)
ORF69	CTAGCTAGCATGTCCATCGATGATTATCG/ CGCGGATCCTTATTCCGGCACAGCCGTAGC	1–4188	1.0
ORF69(595–1395)	CTAGCTAGCGAGGAAGGATGGGGCTGG/ CGCGGATCCTTATTCCGGCACAGCCGTAGC	1786–4188	1.0
ORF69(795–1395)	CTAGCTAGCGTTGGTTCCAGCCCTGTAA/ CGCGGATCCTTATTCCGGCACAGCCGTAGC	2386–4188	0.6
ORF69(995–1395)	CTAGCTAGCCCGCCTACGGGTGTG/ CGCGGATCCTTATTCCGGCACAGCCGTAGC	2986–4188	0.6
ORF69(1195–1395)	CTAGCTAGCAACCCCATGGATGACAT/ CGCGGATCCTTATTCCGGCACAGCCGTAGC	3586–4188	0.8
ORF69(795–1195)	CTAGCTAGCGTTGGTTCCAGCCCTGTAA/ CGCGGATCCTTACTCAGGAGGAATCGGTTC	2386–3585	0.8
ORF69(795–1095)	CTAGCTAGCGTTGGTTCCAGCCCTGTAA/ CGCGGATCCTTAGACCAGATGCTTACCCGTCAC	2386–3285	0.4


*Underlining indicates endonuclease restriction sites.*

### Expression and Purification of phiAxp-3 TSP Variants

pET28a containing cloned TSP genes from phiAxp-3 was transformed into *E. coli* BL21 (»DE3) cells and then cultured in high-salinity liquid LB medium (10 g/L of NaCl) supplemented with 0.1% ampicillin (37°C, 180 rpm). When the OD_600 nm_ reached 0.8, IPTG solution (**Table 1**) was added and then cell was cultured overnight (25°C, 100 rpm).

Bacteria were collected by centrifugation and washed three times with by 0.5× phosphate-buffered saline (PBS). After resuspension in 15 mL lysis buffer, the bacteria were treated by 30% ultrasonic power (Sonics VCX750, United States) to break the cells (2 s on/4 s off) for 10 min. Then, 20 μL of RNase and DNase were added at room temperature for 1 h, and then mixture was centrifuged at 10,000 *g*/min for 40 min at 4°C. The supernatant was collected and purified using a His-tagged-protein Purification Kit (Beijing CWBIO Company) by following the manufacturer’s instructions. Proteins before and after purification were analyzed by SDS-PAGE. The purified proteins were quantified with a Protein Quantification Kit (Beijing BIOMED Company).

### Blockage of Phage Adsorption by Purified TSP Variants

Fresh *A. xylosoxidans* A22732 solution (50 μL, OD_600 nm_ = 3.0) and 150 μg/mL TSP variants solution (50 μL) were mixed at 180 rpm for 10 min at 25°C. Then, 100 μL of 10^3^ plaque-forming units (PFU)/mL phage were added and mixed at 16,000 rpm for 3 min at 4°C. The phage titer in the supernatant (residual PFU) was determined by counting the bacteriophage plaques on *A. xylosoxidans* A22732. Addition of 100 μL 1× PBS (instead of TSP) was used as the negative control, and addition of 100 μL 10^3^ PFU/mL phage was used as the positive control. The phage titer of the positive control was defined as 100% (Kiljunen et al., 2011). The titer was determined using tested phage suspension and agar overlay technique (Carlson, 2005). Each index was tested three times.

### Lipopolysaccharide Extraction

Fresh *A. xylosoxidans* A22732 solution grown overnight at 37°C was centrifuged at 8000 *g*/min for 2 min at 4°C, cells were killed with 0.5% phenol, and the mixture was centrifuged again at 39000 rpm. The bacterial pellet was washed three times with distilled water, lyophilized, treated with 90% phenol/water (1:1), and heated to 65°C. *A. xylosoxidans* A22732 LPS was extracted for 15 min according to previously published methods (Westphal, 1965). The extract was cooled to 4°C, centrifuged at 5000 *g*/min for 30 min, and the aqueous phase was collected. Distilled water was added to the remaining phenol phase and the extraction process was repeated. LPS was purified according to the method of Simin et al. (2011). DNase (20 μg/mL) and RNase (40 μg/mL) were added to the extraction and tubes were kept at 37°C for 4 h. Proteinase K (100 μg/mL) was added to the tubes at 65°C for 1 h, and then they were heated at 100°C for 10 min. The tubes were centrifuged at 8500 *g*/min for 15 min. Supernatants were transferred to 15-mL centrifuge tubes, and two volumes of acetone were added to the extracts. Samples were stored at 4°C overnight to precipitate LPS. The samples were then centrifuged at 2000 *g*/min for 10 min at 4°C, and the precipitates were resuspended in 1 mL distilled water. Extensive dialysis against double distilled water at 4°C was performed until residual phenol was eliminated. Finally, the purified *A. xylosoxidans* A22732 LPS was lyophilized, weighed, and stored at 4°C.

### Enzyme-Linked Immunosorbent Assay (ELISA) Verification of the Interaction Between LPS and phiAxp-3 TSP Variants

The interaction between *A. xylosoxidans* A22732 LPS and TSP variants of phiAxp-3 was tested by ELISA (Martínez-Sernández and Ubeira, 2014). *A. xylosoxidans* LPS was diluted to 100 ng/mL with buffer containing (per L) 1.6 g Na_2_CO_3_ and 2.9 g NaHCO_3_. Then, 100 μL of LPS solution was immobilized on an ELISA plate and incubated at 37°C for 2 h. The mixture was rinsed with PBS-TW2 (PBS solution with 0.05% Tween 20). Bovine serum albumin (BSA) diluted in PBS-TW2 was then added at a final concentration of 0.1% and the mixture was left overnight at 4°C. The wells were then rinsed twice with PBS-TW2. *E. coli* 0111:B4 LPS was used as a negative control. Different concentrations of His-tagged proteins (1 μg/mL – 0.12207 pg/mL) were added (100 μL/well) and the plates were incubated for 30 min at 37°C. The unrelated protein His-tagged fucosidase (from *Bifidobacterium longum* XY01) was used as a negative control. The plate was washed five times with PBS-TW2. In this step, proteins that could not bind to LPS were removed. HRP-conjugated anti-6× His antibody (diluted 1/1,000 in PBS-TW2) was added (100 μL/well) and incubated for 20 min at 37°C. The plate was washed again with PBS-TW2 and incubated for 10 min at room temperature with 50 μL/well SigmaFast OPD substrate before the reaction was stopped by addition of 50 μL of 3 M H_2_SO_4_. The optical density (OD) was measured at 450 nm, and each index was measured three times.

### Effects of Different pH Values and Temperatures on the TSP of phiAxp-3

To explore the effects of pH on the TSP, HCl/NaOH were added to PBS buffer to adjust the pH between 4 and 10. TSP variants were pretreated for 10 min at different pHs and different temperatures (4–70°C, in a water bath). Then, the phage adsorption blockage assay was performed to examine the functionality of the treated TSP.

### *In Vitro* Cell Toxicity of the phiAxp-3 TSP

The toxicity of the TSP of phiAxp-3 (and variants) was tested using normal human liver cells (LO2; American Type Culture Collection). LO2 cells were cultured in high-glucose complete Dulbecco’s modified Eagle’s medium containing 10% fetal calf serum, 1% penicillin (100 U/mL), and streptomycin (100 mg/mL) at 37°C in 5% a CO_2_ atmosphere. Subculture was performed every 4 days (until the cell fusion rate reached 80%). One hundred μL of protein solution diluted in DPBS was added in the following amounts: 12, 6, 3, 1.5, 0.75, or 0.375 μg. In the control group, 100 μL DPBS were added. The survival rate of LO2 cells was tested using the 3-(4,5)-dimethylthiahiazo (-z-y1)-3,5-di- phenytetrazoliumromide (MTT) method after 24 h of culture (Gerlier and Thomasset, 1986). Each index was measured three times.

### Accession Number of Nucleotide Sequence

The amino acid sequence of the TSP of phiAxp-3 was obtained from the NCBI database (accession number ALA45538.1). The accession number of the amino acid sequence of N4 phage gp65 in NCBI is YP_950543.

### Statistical Analyses

Nucleotide sequences were compared using DNAMAN software 8.0 (Lynnonon Biosoft, Quebec City, QC, Canada). The mean values of triplicate experimental readings were determined and subjected to ANOVA. The means were separated using Duncan’s multiple range test with the aid of SPSS version 19.0 (SPSS, Inc., United States).

## Results

### Expression and Purification of TSP and Variants of phiAxp-3

**FIGURE 1:**
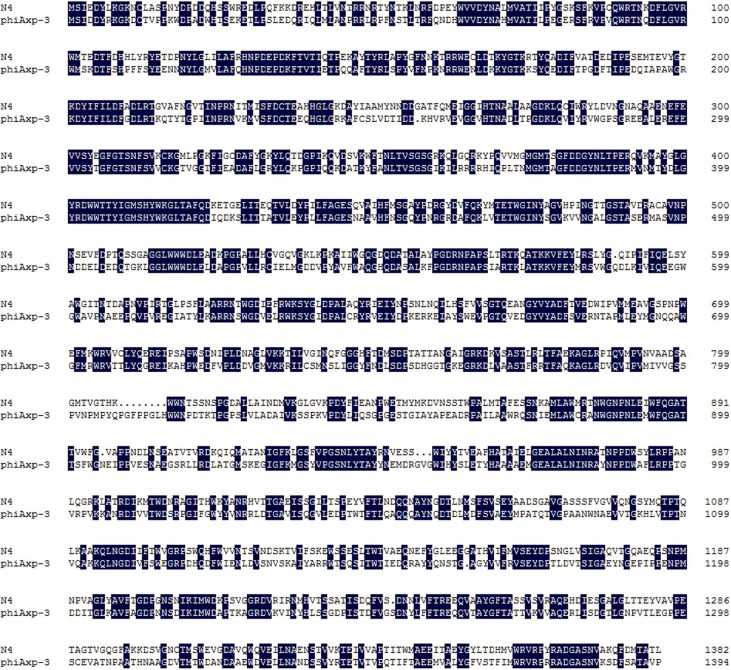
Sequence alignment of the tail proteins (TSP) of phages phiAxp-3 and N4. Identical residues are shaded in black. Gaps (indicated by dotted lines) were introduced into the sequences to maximize the alignment. Numbering is from the N-terminal methionine. Multiple sequence alignment was analyzed and edited with DNAMAN software.

**FIGURE 2:**
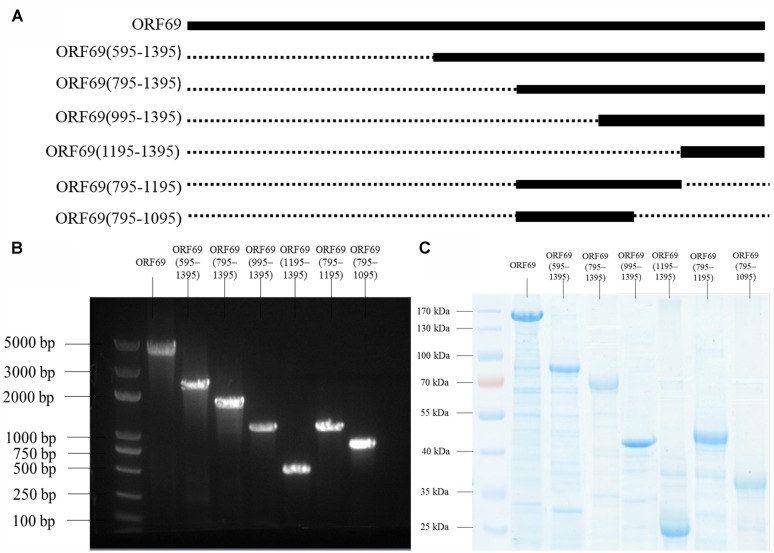
**(A)** Positions of the fragments of the phiAxp-3 TSP studied in this work. The thick lines represent the fragments construced/tested, and the numbers denote the starting and ending amino acid positions. **(B)** Seven fragments spanning different portions of the TSP were expressed as His_6_-tagged fusion proteins: ORF69(full-length), ORF69(595–1395), ORF69(795–1395), ORF69(995–1395), ORF69(1195–1395), ORF69(795–1195), and ORF69(795–1095). **(C)** SDS-PAGE showing the purified TSP variants with molecular masses ranging from 22 to 154 kDa.

The TSP of phiAxp-3 was predicted to be the 69th open reading frame (ORF), a 1395 amino acid protein which shares 54% identity with the amino acid sequence of gp65 of the N4 phage (**Figure 1**). To analyze the location(s) of the receptor-binding region of the TSP, *E. coli* BL21(»DE3) cells were transformed with pET28a expression plasmids that expressed His-tagged full-length ORF69, ORF69(595–1395), ORF69(795–1395), ORF69(995–1395), ORF69(1195–1395), ORF69(795–1195), or ORF69(795–1095) (**Figures 2A,B**). In this notation, for example, ORF69(595–1395) represents amino acids 595 to 1395 of the phiAxp-3 TSP of ORF69. These proteins were then purified by Ni-affinity chromatography with the expected sizes: 154, 88, 66, 44, 22, 44, and 33 kDa, respectively (**Figure 2C**).

### Blockage of phiAxp-3 Adsorption by Purified TSP and Variants

**FIGURE 3:**
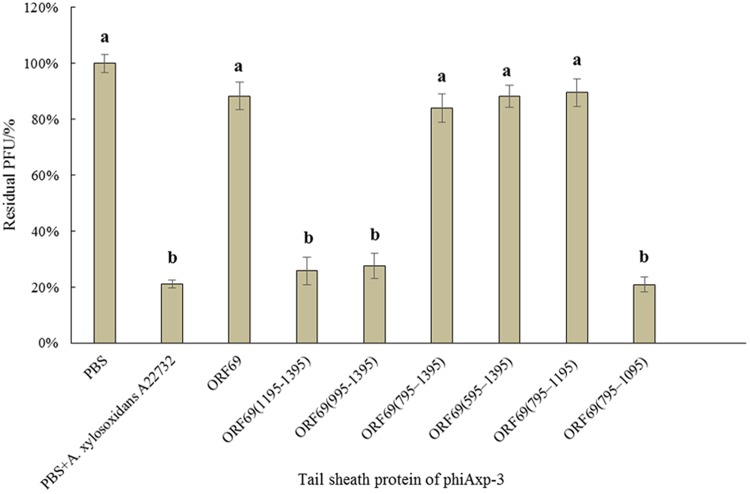
Effects of purified TSP and truncations on phiAxp-3 adsorption to the host bacterium *Achromobacter xylosoxidans* A22732. Numerical values represent the mean of three parallel test results and different letters represent significant differences between data (*P* < 0.05).

We hypothesized that the TSP of phiAxp-3 plays a role in receptor recognition. Therefore, it was necessary to test whether the purified TSP and truncations could inhibit phiAxp-3 binding to the host bacterium. The purified proteins were mixed with fresh *A. xylosoxidans* A22732 solution and the plaquing efficiency of phiAxp-3 was measured. TSP, variants ORF69 (i.e., the full-length protein), ORF69(595–1395), ORF69(795–1395), and ORF69(795–1195) could compete with phage phiAxp-3 for adsorption to the host bacteria, thereby inhibiting phage adsorption (**Figure 3**), but the other TSP truncations ORF69(995–1395), ORF69(1195–1395) and ORF69(795–1095), could not (*P* < 0.05). The adsorption assay demonstrated that TSP functions as a receptor-binding protein, and that residues 795 to 1195 of TSP were sufficient to inhibit phiAxp-3 binding to the receptor *in vitro*.

### Binding of TSP and Variants to *A. xylosoxidans* LPS *in Vitro*

**FIGURE 4:**
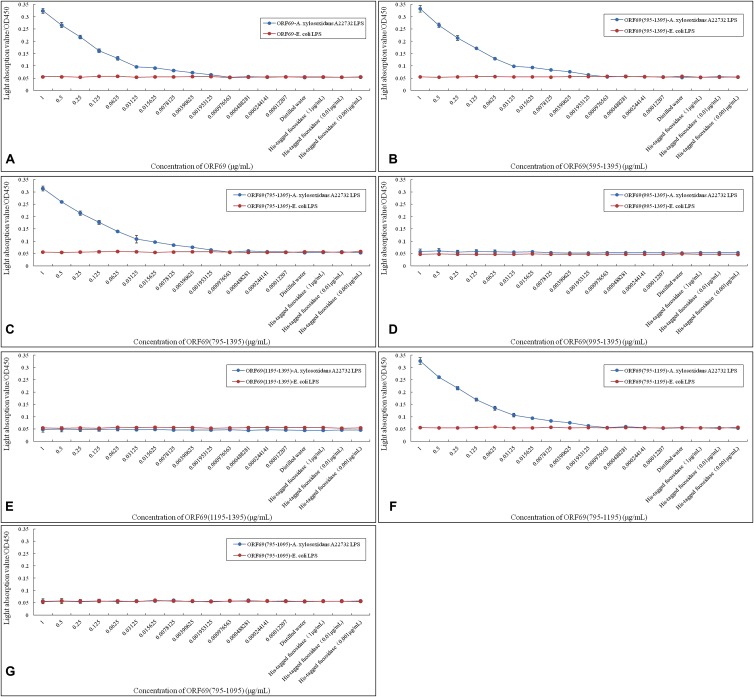
Enzyme-linked immunosorbent assay assessing *A. xylosoxidans* lipopolysaccharide (LPS) interaction with phiAxp-3 TSP and variants. **(A–G)** Represent the *A. xylosoxidans* LPS interaction with ORF69, ORF69(595–1395), ORF69(795–1395), ORF69(995–1395), ORF69(1195–1395), ORF69(795–1195), ORF69(795–1095), respectively.

A previous report demonstrated that LPS is the adsorption target (receptor) of phage phiAxp-3 (Ma et al., 2016). The specific interaction of LPS purified from *A. xylosoxidans* with purified TSP and its variants was confirmed by ELISA (**Figure 4**). ORF69(595–1395) (**Figure 4B**), ORF69(795–1395) (**Figure 4C**) and ORF69(795–1195) (**Figure 4F**), like the ORF69 (**Figure 4A**), interacted specifically with *A. xylosoxidans* LPS and the results revealed a direct correlation between the concentration of each of these TSP variants and the OD in the ELISA. The other three proteins, ORF69(995–1395) (**Figure 4D**), ORF69(1195–1395) (**Figure 4E**) and ORF69(795–1095) (**Figure 4G**), did not interact with *A. xylosoxidans* LPS. Therefore, consistent with the results of the phage adsorption assay (**Figure 3**), residues 795 to 1195 of the TSP are the key region for binding to the receptor, i.e., *A. xylosoxidans* LPS.

### Effects of Different pHs and Temperatures on the TSP of phiAxp-3

**FIGURE 5:**
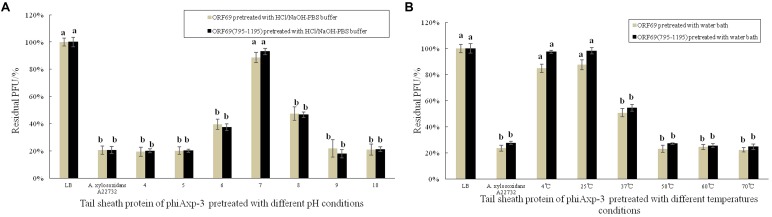
Effects of different pH and temperature pretreatments on the activity of the TSP of phiAxp-3. **(A)** pH group; **(B)** temperature group. Numerical values represent the mean of three parallel test results and different letters represent significant differences between data (*P* < 0.05).

Stability studies of ORF69 and ORF69(795–1195) were conducted at different pH and temperature values. **Figure 5** summarizes the results. The optimum pH for the protein function (i.e., binding to the host bacterium) was 7 (**Figure 5A**), and the optimum temperatures were between 4 and 25°C (**Figure 5B**). The proteins could not effectively compete with phage to adsorb to host bacteria after treatment in acidic (pH 4) or basic (pH 10) conditions.

### Safety Testing of TSP on Cell *in Vitro* Cell Cultures

**FIGURE 6:**
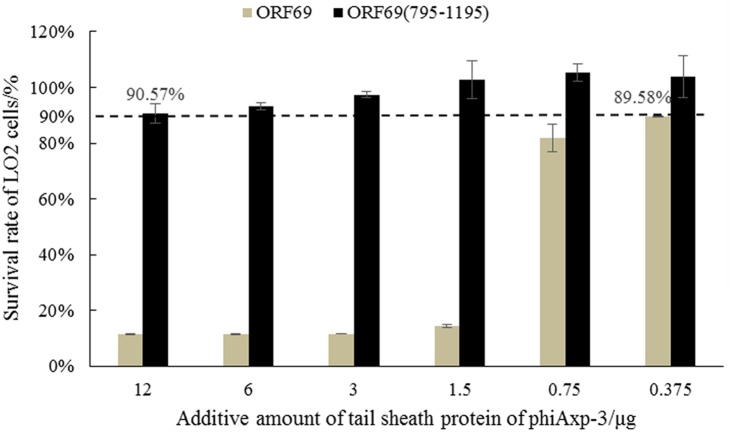
MTT test on survival rates of LO2 cells treated with ORF69 and ORF69(795–1195).

ORF69 and ORF69(795–1195) were tested on the human immortalized normal hepatocyte cell line LO2 *in vitro* to evaluate their direct toxic effects on cells. Cell viability was assessed by the MTT method. ORF69 was toxic to the cell line when applied with protein doses >1.5 μg/well in cultures, however, no toxic or antiproliferative effects of ORF69(795–1195) were observed (**Figure 6**). This result indicates that the cytotoxic region of the TSP is independent of residues 795 to 1195.

## Discussion

Phage phiAxp-3 was isolated from hospital wastewater and specifically lysed *A. xylosoxidans* A22732. phiAxp-3 is an N4-like phage according to morphological study and genome sequencing. However, this novel phage has a different recognition receptor from N4 phage: N4 phage employs the outer membrane protein NfrA as the recognition receptor, whereas phiAxp-3 uses LPS (Ma et al., 2016). Gp65 of N4, encoding the TSP, is thought to be the receptor-binding protein. Using the amino acid sequences of TSP of phage N4 as blast query against NCBI database, there is no recognizable sequence similarity between N4 and other reported N4 like phages. This protein was highly unique, the most similar homology searched was the TSP (encoded by ORF69) of phiAxp-3, although they share only 54% amino acid identity and their binding receptors were totally different (outer membrane protein and LPS, respectively). More than 30 N4-like phages have been reported and sequenced, their most notable feature is the giant RNA polymerase carried in the capsid. The tail proteins (such as tail sheath protein, tail fiber protein, or tail spike protein) responsible for receptor binding showed extensive sequence variability among sequenced N4-like phages. Tail proteins of phages are considered to be a major factor in host specificity. This might be one of the reasons why the hosts of N4-like phages are diverse. A previous study showed that 25 N4-like phage genomes share 14 conserved core genes, which do not contain any phage binding genes (Chan et al., 2014), the highly unique binding proteins imply a difference in receptor recognition.

In the present study, recombinant full-length TSP (ORF69) of phiAxp-3 and six variants were co-incubated with the host bacterium. The truncated protein ORF69(795–1195) could directly bind to the host bacteria, with the same function as ORF69 (**Figure 3**). ELISA confirmed that ORF69(795–1195) could interact directly with *A. xylosoxidans* A22732 LPS *in vitro* (**Figure 4F**). Brzozowska et al. (2015) demonstrated that the T4 bacteriophage tail fiber adhesin for specific LPS recognition is localized near the C-terminus of the protein. In the present study, the essential sequence of phiAxp-3 TSP for specific LPS recognition was also localized near the C-terminus of ORF69. Short proteins recognizing bacterial cells in a specific manner have major implications for better understanding the molecular recognition of cell surfaces and for bacteria detection (Brzozowska et al., 2015).

The characterization of potential therapeutic phages or phage preparations should be undertaken. It is necessary to confirm the stability of the therapeutic phages or phage preparations in different physicochemical conditions (Jończyk et al., 2011). Therefore, in this study, stability studies of ORF69 and ORF69(795–1195) were conducted at different pHs and temperatures. Phages may provide good alternatives to conventional antibiotics, especially for treatment of antibiotic-resistant pathogenic bacteria (Magiorakos et al., 2012; Deris et al., 2013). Weber-Dąbrowska et al. (2016) propounded that the therapeutic phages or phage preparations were non-toxic, and other researchers support this view in a recent report (Jończyk-Matysiak et al., 2017).

Almost all phages recognize receptors through the phage tail (Chaturongakul and Ounjai, 2014). Therefore, the characteristics, especially the safety, of tail proteins of phages must be determined. Miernikiewicz et al. (2016) demonstrated that the tail protein gp12 of T4 phage is non-toxic. In the present study, the toxicities of ORF69 of phiAxp-3 and ORF69(795–1195) were evaluated using LO2 cells. The survival rate of LO2 cells decreased as the dose of ORF69 (0.375–12 μg/well in cultures) increased, indicating that ORF69 may be toxic to the cells (Berridge and Tan, 1993). However, given the same amount of ORF69(795–1195), the survival rate of LO2 cells was >90% (**Figure 6**), indicating that ORF69(795–1195) is non-toxic in this range (doses: 0.375–12 μg/well in cultures).

In this study, ORF69(795–1195) was shown to interact with *A. xylosoxidans* LPS. Recombinant gp12 (the T4 phage tail adhesin) retains the ability to bind LPS, and gp12 was successfully used in an *in vivo* experiment to counteract LPS-induced inflammation in rice (Miernikiewicz et al., 2016). Kim et al. (2017) demonstrated that a non-toxic LPS binding peptide was an effective anti-inflammatory peptide for the treatment of acute lung injury. Brackett et al. (1997) demonstrated that a synthetic peptide corresponding to amino acid residues 20 through 44 of the neutrophil-derived 37-kDa cationic antimicrobial protein (CAP37 P20–44) can bind lipid A of LPS, which could be useful in attenuating *in vivo* responses induced during endotoxemia, including sepsis. The emergence of antibiotic-resistant bacteria can cause serious clinical and public health problems (Jung et al., 2017). N4-like phages have been used as therapeutic agent in phage therapy against *Pseudomonas aeruginosa* infections and have been shown to be safe and effective (Shigehisa et al., 2016). Characterization of the TSP of phage phiAxp-3 might expand our knowledge of using phages as an alternative agent to control multidrug-resistant bacteria. As LPS binding proteins/peptides successfully prevented endotoxin-induced responses *in vivo*, ORF69(795–1195) may be used to develop potential drugs for clinical treatment.

## Conclusion

In this study, genes for the TSP of the phiAxp-3 and truncated variants were shown to inhibit phiAxp-3 adsorption onto the host bacterial surface. Experimental results showed no significant difference in residual PFU between the experimental groups [ORF69(595–1395), ORF69(795–1395), and ORF69(795–1195)] and the control (ORF69). Similar experimental results were observed in the interaction between the TSP variants and LPS from the host bacterium. Therefore, proteins with similar functions to full-length TSP (ORF69) were screened and the shortest was identified: ORF69(795–1195). The *in vitro* cell toxicity of ORF69 and ORF69(795–1195) was tested; ORF69 was highly toxic, but the ORF69(795–1195) was non-toxic when at dose ranges are 0.375–12 μg. These experimental results can provide references for the application of the TSP of phiAxp-3.

## Author Contributions

ZZ and XZ designed the research. ZZ and JZ performed the research. JY and AJ contributed the new reagents or analytic tools. XC and RF analyzed the data. XW, HL, WL, and WY provided experimental materials and equipments. ZZ and CT wrote the paper.

## Conflict of Interest Statement

The authors declare that the research was conducted in the absence of any commercial or financial relationships that could be construed as a potential conflict of interest.

## References

[B1] Berridge M. V. Tan A. S. (1993). Characterization of the cellular reduction of 3-(4,5-dimethylthiazol-2-yl)-2,5-diphenyltetrazolium bromide (MTT): subcellular localization, substrate dependence, and involvement of mitochondrial electron transport in MTT reduction. *Arch. Biochem. Biophys.* 303 474–482. 10.1006/abbi.1993.1311 8390225

[B2] Brackett D. J. Lerner M. R. Lacquement M. A. He R. Pereira H. A. (1997). A synthetic lipopolysaccharide-binding peptide based on the neutrophil-derived protein CAP37 prevents endotoxin-induced responses in conscious rats. *Infect. Immun.* 65 2803–2811. 9199453 10.1128/iai.65.7.2803-2811.1997 PMC175395

[B3] Brzozowska E. Pyra A. Miśków M. Górska S. Gamian A. (2015). C-terminal sequence determinants of T4 bacteriophage tail fiber adhesin for specific lipopolysaccharide recognition. *Symbiosis* 3 1–5. 10.15226/sojmid/3/1/00130

[B4] Carlson K. (2005). “Working with bacteriophages: common techniques and methodological approaches,” in *Bacteriophages: Biology and Applications*, eds Kutter E. Sulakvelidze A. (Boca Raton, FL: CRC Press), 437–494.

[B5] Chan J. Z. Millard A. D. Mann N. H. Schafer H. (2014). Comparative genomics defines the core genome of the growing N4-like phage genus and identifies N4-like Roseophage specific genes. *Front. Microbiol.* 5:506. 10.3389/fmicb.2014.00506 25346726 PMC4193335

[B6] Chaturongakul S. Ounjai P. (2014). Phage–host interplay: examples from tailed phages and Gram-negative bacterial pathogens. *Front. Microbiol.* 5:442 10.3389/fmicb.2014.00442 PMC4138488 25191318

[B7] Chen Z. Fang H. Wang L. Sun F. Wang Y. Yin Z. (2014). IMP-1 encoded by a novel *Tn402*-like class 1 integron in clinical *Achromobacter xylosoxidans*, China. *Sci. Rep.* 4:7212. 10.1038/srep07212 25428613 PMC4245530

[B8] Choi K. H. Mcpartland J. Kaganman I. Bowman V. D. Rothman-Denes L. B. Rossmann M. G. (2008). Insight into DNA and protein transport in double-stranded DNA viruses: the structure of bacteriophage N4. *J. Mol. Biol.* 378 726–736. 10.1016/j.jmb.2008.02.059 18374942 PMC2396777

[B9] Deris J. B. Kim M. Zhang Z. Okano H. Hermsen R. Groisman A. (2013). The innate growth bistability and fitness landscapes of antibiotic-resistant bacteria. *Science* 342:1237435. 10.1126/science.1237435 24288338 PMC4059556

[B10] Durán A. E. Muniesa M. Méndez X. Valero F. Lucena F. Jofre J. (2002). Removal and inactivation of indicator bacteriophages in fresh waters. *J. Appl. Microbiol.* 92 338–347. 10.1046/j.1365-2672.2002.01536.x 11849363

[B11] Gerlier D. Thomasset N. (1986). Use of MTT colorimetric assay to measure cell activation. *J. Immunol. Methods* 94 57–63. 10.1016/0022-1759(86)90215-2 3782817

[B12] Hodyra-stefaniak K. Miernikiewicz P. Drapała J. Drab M. Jończykmatysiak E. Lecion D. (2015). Mammalian Host-Versus-Phage immune response determines phage fate *in vivo*. *Sci. Rep.* 5:14802. 10.1038/srep14802 26440922 PMC4594097

[B13] Jończyk E. Kłak M. Międzybrodzki R. Górski A. (2011). The influence of external factors on bacteriophages–review. *Folia Microbiol.* 56 191–200. 10.1007/s12223-011-0039-8 21625877 PMC3131515

[B14] Jończyk-Matysiak E. Weber-Dąbrowska B. Owczarek B. Międzybrodzki R. Łusiak-Szelchowska M. Łodej N. (2017). Phage-phagocyte interactions and their implications for phage application as therapeutics. *Viruses* 9:E150. 10.3390/v9060150 28613272 PMC5489797

[B15] Jung L. S. Ding T. Ahn J. (2017). Evaluation of lytic bacteriophages for control of multidrug-resistant *Salmonella typhimurium*. *Ann. Clin. Microbiol. Antimicrob.* 16:66. 10.1186/s12941-017-0237-6 28938899 PMC5610459

[B16] Kiino D. R. Rothmandenes L. B. (1989). Genetic analysis of bacteriophage N4 adsorption. *J. Bacteriol.* 171 4595–4602. 10.1128/jb.171.9.4595-4602.1989 2670887 PMC210256

[B17] Kiino D. R. Singer M. S. Rothmandenes L. B. (1993). Two overlapping genes encoding membrane proteins required for bacteriophage N4 adsorption. *J. Bacteriol.* 175 7081–7085. 10.1128/jb.175.21.7081-7085.1993 8226649 PMC206836

[B18] Kiljunen S. Datta N. Dentovskaya S. V. Anisimov A. P. Knirel Y. A. Bengoechea J. A. (2011). Identification of the lipopolysaccharide core of *Yersinia pestis* and *Yersinia pseudotuberculosis* as the receptor for bacteriophage φA1122. *J. Bacteriol.* 193 4963–4972. 10.1128/JB.00339-11 21764935 PMC3165662

[B19] Kim J. Y. Piao C. Kim G. Lee S. Lee M. S. Jeong J. H. (2017). Combined delivery of a lipopolysaccharide-binding peptide and the heme oxygenase-1 gene using deoxycholic acid-conjugated polyethylenimine for the treatment of acute lung injury. *Macromol. Biosci.* 17:1600490. 10.1002/mabi.201600490 28508430

[B20] Krüger D. H. Schroeder C. (1981). Bacteriophage T3 and bacteriophage T7 virus-host cell interactions. *Microbiol. Rev.* 45 9–51. 6261110 10.1128/mr.45.1.9-51.1981 PMC281497

[B21] Lindberg A. A. (1973). Bacteriophage receptors. *Ann. Rev. Microbiol.* 27 205–241. 10.1146/annurev.mi.27.100173.001225 4584686

[B22] Ma Y. Li E. Qi Z. Li H. Wei X. Lin W. (2016). Isolation and molecular characterisation of *Achromobacter* phage phiAxp-3, an N4-like bacteriophage. *Sci. Rep.* 6:24776. 10.1038/srep24776 27094846 PMC4837373

[B23] Magiorakos A. P. Srinivasan A. Carey R. B. Carmeli Y. Falagas M. E. Giske C. G. (2012). Multidrug-resistant, extensively drug-resistant and pandrug-resistant bacteria: an international expert proposal for interim standard definitions for acquired resistance. *Clin. Microbiol. Infec.* 18 268–281. 10.1111/j.1469-0691.2011.03570.x 21793988

[B24] Martínez-Sernández V. Ubeira F. M. (2014). Competitive ELISA for protein-lipopolysaccharide (LPS) binding. *Bioprotocol* 4:e1298.

[B25] Mcpartland J. Rothman-Denes L. B. (2009). The tail sheath of bacteriophage N4 interacts with the *Escherichia coli* receptor. *J. Bacteriol.* 191 525–532. 10.1128/JB.01423-08 19011026 PMC2620810

[B26] Miernikiewicz P. Kłopot A. Soluch R. Szkuta P. Kęska W. Hodyra-Stefaniak K. (2016). T4 phage tail adhesin gp12 counteracts LPS-induced inflammation *in vivo*. *Front. Microbiol.* 7:1112. 10.3389/fmicb.2016.01112 27471503 PMC4943950

[B27] Park K. Myung H. (2014). Observation of inflammatory responses in mice orally fed with bacteriophage T7. *J. Appl. Microbiol.* 117 627–633. 10.1111/jam.12565 24916438

[B28] Reardon S. (2017). Modified viruses deliver death to antibiotic-resistant bacteria. *Nature* 546 586–587. 10.1038/nature.2017.22173 28661508

[B29] Richter Ł. Janczuk-Richter M. Niedziółka-Jönsson J. Paczesny J. Holyst R. (2017). Recent advances in bacteriophage-based methods for bacteria detection. *Drug Discov. Today* 23 448–455. 10.1016/j.drudis.2017.11.007 29158194

[B30] Sader H. S. Jones R. N. (2005). Antimicrobial susceptibility of uncommonly isolated non-enteric Gram-negative bacilli. *Int. J. Antimicrob. Agents* 25 95–109. 10.1016/j.ijantimicag.2004.10.002 15664479

[B31] São-José C. Baptista C. Santos M. A. (2004). *Bacillus subtilis* operon encoding a membrane receptor for bacteriophage SPP1. *J. Bacteriol.* 186 8337–8346. 10.1128/JB.186.24.8337-8346.2004 15576783 PMC532427

[B32] São-José C. Lhuillier S. Lurz R. Melki R. Lepault J. Santos M. A. (2006). The ectodomain of the viral receptor YueB forms a fiber that triggers ejection of bacteriophage SPP1 DNA. *J. Biol. Chem.* 281 11464–11470. 10.1074/jbc.M513625200 16481324

[B33] Schofield D. A. Westwater C. (2009). Phage-mediated bioluminescent detection of *Bacillus anthracis*. *J. Appl. Microbiol.* 107 1468–1478. 10.1111/j.1365-2672.2009.04332.x 19426264

[B34] Shigehisa R. Uchiyama J. Kato S. Takemura-Uchiyama I. Yamaguchi K. Miyata R. (2016). Characterization of *Pseudomonas aeruginosa* phage KPP21 belonging to family *Podoviridae* genus N4-like viruses isolated in Japan. *Microbiol. Immunol.* 60 64–67. 10.1111/1348-0421.12347 26616567

[B35] Simin R. Noor A. Bahman T. Mahmood J. T. Omid Z. Reza A. (2011). Extraction, purification and characterization of lipopolysaccharide from *Escherichia coli* and *Salmonella typhi*. *Avicenna J. Med. Biotechnol.* 3 3–9. 23407691 PMC3558174

[B36] Singh A. Arya S. K. Glass N. Hanifi-Moghaddam P. Naidoo R. Szymanski C. M. (2010). Bacteriophage tailspike proteins as molecular probes for sensitive and selective bacterial detection. *Biosens. Bioelectron.* 26 131–138. 10.1016/j.bios.2010.05.024 20541928

[B37] Soffer N. Woolston J. Li M. Das C. Sulakvelidze A. (2017). Bacteriophage preparation lytic for *Shigella* significantly reduces *Shigella sonnei* contamination in various foods. *PLoS One* 12:e0175256. 10.1371/journal.pone.0175256 28362863 PMC5376334

[B38] Weber-Dąbrowska B. Jończyk-Matysiak E. Żaczek M. Łobocka M. Łusiak-Szelachowska M. Górski A. (2016). Bacteriophage procurement for therapeutic purposes. *Front. Microbiol.* 7:1177 10.3389/fmicb.2016.01177 PMC4981656 27570518

[B39] Westphal O. (1965). Bacterial lipopolysaccharides: extraction with phenol-water and further applications of the procedure. *Methods Carbohydr. Chem.* 5 83–92.

